# Effective isolation of cannabidiol and cannabidiolic acid free of psychotropic phytocannabinoids from hemp extract by fast centrifugal partition chromatography

**DOI:** 10.1007/s00216-023-04782-9

**Published:** 2023-06-29

**Authors:** Matej Maly, Frantisek Benes, Zuzana Binova, Marie Zlechovcova, Petr Kastanek, Jana Hajslova

**Affiliations:** 1 Department of Food Analysis and Nutrition, University of Chemistry and Technology, Technická 5, 166 28 Prague 6, Czech Republic; 2 Ecofuel Laboratories s.r.o., Ocelářská 9, 190 00 Prague 9, Czech Republic

**Keywords:** Hemp extract, Single step fractionation, Phytocannabinoids, Fast centrifugal partition chromatography, Pure cannabidiol/cannabidiolic acid, Removing ∆^9^-tetrahydrocannabinol/∆^9^-tetrahydrocannabinolic acid

## Abstract

Cannabidiol (CBD), together with its precursor cannabidiolic acid (CBDA), is the major phytocannabinoid occurring in most hemp cultivars. To ensure the safe use of these compounds, their effective isolation from hemp extract is required, with special emphasis on the elimination of ∆^9^-tetrahydrocannabinol (∆^9^-THC) and ∆^9^-tetrahydrocannabinolic acid (∆^9^-THCA-A). In this study, we demonstrate the applicability of fast centrifugal partition chromatography (FCPC) as a challenging format of counter-current preparative chromatography for the isolation of CBD and CBDA free of psychotropic compounds that may occur in *Cannabis sativa* L. plant extracts. Thirty-eight solvent mixtures were tested to identify a suitable two-phase system for this purpose. Based on the measured partition coefficients (K_D_) and separation factors (α), the two-phase system consisting of n-heptane:ethyl acetate:ethanol:water (1.5:0.5:1.5:0.5; *v:v:v:v*) was selected as an optimal solvent mixture. Employing UHPLC-HRMS/MS for target analysis of collected fractions, the elution profiles of 17 most common phytocannabinoids were determined. Under experimental conditions, the purity of isolated CBD and CBDA was 98.9 and 95.1% (*w/w*), respectively. Neither of ∆^9^-THC nor of ∆^9^-THCA-A were present; only trace amounts of other biologically active compounds contained in hemp extract were detected by screening against in-house spectral library using UHPLC-HRMS.

**Graphical abstract:**

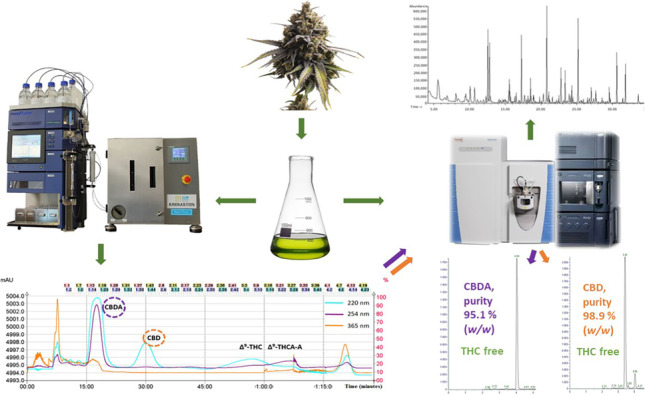

**Supplementary information:**

The online version contains supplementary material available at 10.1007/s00216-023-04782-9.

## Introduction

In recent years, interest in phytocannabinoids, biologically active compounds that occur in *Cannabis sativa* L. plants, has increased enormously. These unique secondary metabolites have the potential to bind to specific endocannabinoid receptors in the human body [1]. Currently, more than 100 different natural compounds with phytocannabinoid-like structures are known [2]; of them, ∆^9^-tetrahydrocannabinol (∆^9^-THC) and its isomer, cannabidiol (CBD), are probably of the highest concern for the scientific community. The concentration of phytocannabinoids and their ratios depend mainly on the respective cultivars and the growing conditions in particular localities. Furthermore, post-harvest processing practices play an important role regarding patterns of phytocannabinoids in the final product. Specifically, elevated temperatures can induce various chemical changes, including decarboxylation of non-psychoactive phytocannabinoid acids such as ∆^9^-tetrahydrocannabinol acid-A (∆^9^-THCA-A) and cannabidiol acid (CBDA), yielding ∆^9^-THC and CBD, respectively. ∆^9^-THC is the key component in some special medicinal preparations; nevertheless, due to its psychotropic activity, the use of products containing this compound, unless they are not prescribed for a specific therapeutic purpose, is illegal in some countries around the world [3]. The same regulation is applied to the ∆^9^-THCA-A, ∆^9^-THC precursor that often occurs in large amounts in some cultivars of *Cannabis sativa* L. The assumed beneficial bioactivities of other major non-psychotropic phytocannabinoids, namely CBD and, more recently, cannabigerol (CBG), have been intensively studied as potential uses in the pharmaceutical, cosmetic, and/or food industry which are foreseen [4, 5]. In 2020, the European Union (EU) supreme court decided that CBD is not a narcotic compound and can be freely traded at EU markets [6]; nevertheless, the discussion about its status as novel foods has not been completed due to various uncertainties and knowledge gaps. In other words, at present, there has been no authorization of CBD, of any other cannabinoids, nor of products containing either CBD and/or other cannabinoids derived from the *Cannabis sativa* L. plant under Regulation (EU) 2015/2283 on novel foods. In any case, CBD is typically isolated from hemp extracts, which commonly contain a number of other secondary plant metabolites, including ∆^9^-THC/∆^9^-THCA-A. Under these conditions, various “impurities” are often present in isolated CBD. It should be noted that even traces of ∆^9^-THC/∆^9^-THCA-A in purified CBD may pose a problem. In 2015, the European Food Safety Authority (EFSA) established an Acute Reference Dose (ARfD) of 1 μg/kg body weight for ∆^9^-THC [7], which, based on our earlier study [8] focused on commercial CBD oils, is not difficult to fulfill, or even exceed, when consuming some products prepared from “natural” hemp extracts.

Currently, both synthetic and “natural” CBDs are available in the grey markets; the latter is commonly obtained from CBD/CBDA-rich hemp cultivars (chemotype III plants with a low THCA/CBDA ratio <  < 1.0) [9]. Various extraction procedures have been developed, including supercritical fluid extraction using carbon dioxide and ethanol-based extraction [10, 11]. Although the primary extraction step is not, in principle, too complicated, the CBD purification procedure might represent a challenge. There are several alternative approaches to remove ∆^9^-THC and its precursor ∆^9^-THCA-A, including selective precipitation, preparative chromatography, and flash chromatography [12, 13]. In any case, the effectiveness and throughput of these processes should be considered under large-scale production conditions. In this context, one of the interesting purification techniques is counter-current chromatography (CCC). In an older study [14] reporting on the use of FCPC for the fractionation of phytocannabinoids contained in a cannabis extract, the neutral and acid forms were first isolated off-line and then separated into two different solvent systems. In the more recent paper [15] employing FCPC, only acidic phytocannabinoids were fractionated, so the process is rather time-consuming. The more recent study introduced an interesting alternative to batch separation [16]. The authors focused on the isolation of minor neutral phytocannabinoids by using trapping multiple dual mode (MDM), a flow-reversal liquid-liquid chromatography operational mode. For each isolated compound, a specific solvent system was used; nevertheless, the needed solvent volumes were reduced compared to batch operation. The purities of the isolated fractions were 93–99%, yields of 73–95% [16].

CCC is a generic term that covers all forms of liquid chromatography (LC) that use two immiscible liquid phases without any solid support [17]. Separation of analytes is based on differences in their partition coefficients (K_D_). Currently, two different technical solutions are the most widely used, hydrodynamic high-speed counter current chromatography (HSCCC) and hydrostatic fast centrifugal partition chromatography (FCPC) [18, 19]. In the latter case, the “stationary phase” is first fed into the rotor, where it is maintained during separation by centrifugal force. The second liquid of the two-phase system, the “mobile phase,” is pumped at high pressure through a rotor filled with the stationary phase. During phase mixing and following decantation, partition of solutes occurs between the two phases. Depending on this process, the analytes are retained in the system for different periods of time, the eluted fractions of the mobile phase are collected at the outlet of the system, and in an ideal case, separation is achieved [17, 19].

Because of the use of a liquid stationary phase, CCC benefits from many advantages over traditional preparative techniques: these include the absence of irreversible adsorption of some molecules contained in the separated sample, high loading capacity, total recovery of injected samples, low risk of sample denaturation, lower solvent consumption, and the absence of an expensive stationary phase. In addition, CCC can be coupled with other on-line separation techniques, and it is possible to use it directly for crude extracts [20–23].

The key objective of the current study that focused on hemp extract was to identify optimal FCPC conditions for obtaining CBD/CBDA-rich fractions free of ∆^9^-THC/∆^9^-THCA-A, i.e., to isolate safe products for further use. In addition, we investigated the possible presence of other (minor) biologically active co-extracts in the targeted compounds obtained within a single-step procedure.

## Experimental

### *Cannabis sativa* L. extract

The hemp extract was kindly supplied by Ecofuel Laboratories (Prague, Czech Republic). Hemp was extracted by supercritical fluid extraction with ethanol as a modifier. Fifty grams of the extract was dissolved in 250 mL of ethanol (purity 96%), winterized at  − 20 °C for 24 h and then filtered. In this way, the waxes and other frozen solids were separated from the liquid phase rich in phytocannabinoids; ethanol was then evaporated using a rotary evaporator. The concentrations of individual phytocannabinoids in the solvent-free extract are summarized in Table 1. The analytical method used for sample analysis is described in paragraph “U-HPLC-HRMS/MS analysis of hemp extract and its fractions.”

**Table 1:** Content of 17 phytocannabinoids in solvent-free hemp extract used for FCPC fractionation

Analytes *	mg/g	% (w/w)
Cannabidiol acid (CBDA)	340	34.0
Cannabichromene acid (CBCA)	68.9	6.9
Cannabidiol (CBD)	60.5	6.1
Delta-9-tetrahydrocannabinol acid (∆^9^-THCA-A)	47.2	4.7
Delta-9-tetrahydrocannabinol (∆^9^-THC)	15.6	1.6
Cannabigerol acid (CBGA)	13.6	1.4
Cannabidivarin acid (CBDVA)	4.9	0.49
Cannabichromene (CBC)	4.1	0.40
Cannabinol acid (CBNA)	2.0	0.20
Cannabinol (CBN)	1.7	0.17
Cannabigerol (CBG)	0.79	0.08
Cannabicyclol acid (CBLA)	0.68	0.07
Tetrahydrocannabivarin acid (THCVA)	0.62	0.06
Cannabidivarin (CBDV)	0.35	0.03
Tetrahydrocannabivarin (THCV)	0.16	0.02
Cannabicyclol (CBL)	0.16	0.02
Delta-8-tetrahydrocannabinol (∆^8^-THC)	< Limit of quantification	< Limit of quantification
Sum of phytocannabinoids	561	56.1

*The molecular structures of the analytes are shown at Figure S2

### Chemicals and materials

LC–MS grade chemicals (methanol, ethyl acetate, isopropanol, ammonium formate, formic acid, n-hexane, n-heptane, n-pentane) with purities in the range of 98.5–99.8% were purchased from Merck. Deionized water (18 mΩ) was obtained from an internal Milli-Q system (Millipore). P.A. 96% ethanol was purchased from Lach-Ner. Analytical standards of 17 phytocannabinoids (cannabidiol (CBD), cannabidiolic acid (CBDA), delta-8-tetrahydrocannabinol (∆^8^-THC), delta-9-tetrahydrocannabinol (∆^9^-THC), delta-9-tetrahydrocannabinolic acid (∆^9^-THCA-A), cannabigerol (CBG), cannabigerolic acid (CBGA), cannabicyclol (CBL), cannabicyclolic acid (CBLA), cannabidivarin (CBDV), cannabidivarinic acid (CBDVA), tetrahydrocannabivarin (THCV), tetrahydrocannabivarinic acid (THCVA), cannabinol (CBN), cannabinolic acid (CBNA), cannabichromene (CBC), and cannabichromenic acid (CBCA)) in purity ranges from 95 to 100% were purchased from Merck.

### Apparatus

Separation was performed using a fast centrifugal partition chromatograph FCPC® A, purchased from the Rousselet-Robatel Kromaton Company (Annonay, France), with a rotor capacity of 1000 mL. The system was equipped with a gradient pump, a Rheodyne valve with a 50 mL sample loop, a DAD detector, and a fraction collector (Kromaton, Annonay, France). Chromatograms were recorded at 220, 254, and 365 nm. All experiments were performed at room temperature (20 ± 2 °C).

For the analysis of phytocannabinoids in experimental samples, we used an ultra-performance liquid chromatograph (U-HPLC) UltiMate 3000 (Thermo Scientific, USA) coupled with a tandem high-resolution mass spectrometer (HRMS/MS) with Orbitrap mass analyzer, Q-Exactive (Thermo Scientific, USA), and an electrospray source (ESI) operated both in positive/negative modes. Detailed analytical conditions are described briefly in paragraph “U-HPLC-HRMS/MS analysis of hemp extract and its fractions”; they were similar to those reported in our earlier paper [24].

### Determination of partition coefficients (K_D_) and separation factor (α)

In total, 38 solvent mixtures, listed in Table 2, were tested to determine the partition coefficients of the individual phytocannabinoids in a two-phase system. K_D_s were calculated using the following equation [19, 25]:

$$K_D=\frac{{\left[A\right]}_{upper\;phase}}{{\left[A\right]}_{lower\;phase}}$$

where *[A]* is the concentration of analyte A in its respective phase. Based on the results, the separation factors were calculated for the selected solvent system and for the selected analytes (∆^9^-THC, ∆^9^-THCA-A, CBD, CBDA) using the following equation [25]:

$$\alpha =\frac{{K}_{2}}{{K}_{1}};({K}_{2}>{K}_{1})$$

**Table 2:** Thirty-eight tested solvent systems for the selection of optimal FCPC mobile and stationary phases. The composition of solvent systems 20–38 was identical to systems 1–19, with the addition of 0.1% formic acid. The experimental K_D_ values for solvent systems 1–38 are summarized in Table S1

Solvent system	n-Heptane	n-Hexane	n-Pentane	Ethyl acetate	Ethanol	Methanol	Water*
1	0.5			1.5		0.5	1.5
2	1			1		1	1
3	1.5			0.5		1.5	0.5
4	2					2	
5	0.5			1.5	0.5		1.5
6	1			1	1		1
7	1.5			0.5	1.5		0.5
8		0.5		1.5	0.5		1.5
9		1		1	1		1
10		1.5		0.5	1.5		0.5
11		1.8		0.2		1.8	0.2
12		1.2		0.8		1.2	0.8
13		1		1		1	1
14		0.8		1.2		0.8	1.2
15		1.6		0.4	1		1
16			0.5	1.5		0.5	1.5
17			1	1		1	1
18			1.5	0.5		1.5	0.5
19				2			2

*Deionized water was used

The experimental procedure was as follows: an aliquot of hemp extract (10 mg) was weighed in a 20 mL glass tube, and 4 mL of the selected solvent system mixture (the ratios of individual components shown in Table 2) were added to the sample. The tube was then tightly closed and vigorously shaken for 30 s. After separation of the two phases (t < 30 s), a 100 μL aliquot of each layer after separation was removed and transferred to a vial, diluted with 900 μL of ethanol and analyzed by U-HPLC-HRMS. It should be noted that in solvent systems containing ethyl acetate and water, slow hydrolysis of ethyl acetate may occur over the time. This problem was pointed out by Berthod et al. [26]; nevertheless, the authors concluded that this pH lowering due to the release of acetic acid becomes significant after several days to weeks of storage, depending on the composition of the solvent system. To prevent undesirable extent of ethyl acetate hydrolysis, all solvents systems used in our experiments were freshly prepared.

### Preparation of the tested solvent system and hemp extract for fractionation

Based on the calculated partition coefficients K_D_ and separation factors α, summarized in Table S1 and Table 3, the biphasic solvent systems 7 and 29, consisting of [7] n-heptane/ethyl acetate/ethanol/water (1.5:0.5:1.5:0.5, *v/v/v/v*) and [29] n-hexane/ethyl acetate/ethanol/water (1.5:0.5:1.5:0.5, *v/v/v/v*) + 0.1% formic acid, were chosen for follow-up experiments. The solvent mixture (750 mL of n-heptane/n-hexane, 250 mL of ethyl acetate, 750 mL of ethanol and 250 mL of deionized water) was shaken vigorously in a 2000 mL separatory funnel and then allowed to separate. The upper phase of the biphasic system was used as a stationary phase, while the lower aqueous layer was the mobile phase (i.e., descending mode was used). The sample solution was prepared by dissolving 10 g of winterized hemp extract in the upper organic phase and diluting it to 50 mL in a volumetric flask.

**Table 3:** Calculated separation factors (α) for CBD, CBDA, Δ^9^-THC, and Δ^9^-THCA-A in selected solvent systems 7 and 29 (described in Table 2)

Solvent system 7		CBDA	CBD	Δ^9^-THCA-A	Δ^9^-THC
Separation factor α	CBDA	X	1.6	2.4	3.5
	CBD	1.6	X	1.5	2.1
Solvent system 29		CBDA	CBD	Δ^9^-THCA-A	Δ^9^-THC
Separation factor α	CBDA	X	1.7	2.6	3.6
	CBD	1.7	X	1.6	2.1

### FCPC fractionation procedure

Keeping the rotor speed at 600 rpm and the flow rate at 125 mL/min, the “column” (rotor) was first filled with the upper stationary phase (i.e., descending mode was chosen). After 13 min, the rotor speed was increased to 1600 rpm, and the lower aqueous phase was introduced into the rotor as the mobile phase with a flow rate of 50 mL/min. The back pressure increased from 15 to 78 bars, causing a displacement of about 23% of the stationary phase for solvent system 7 (for solvent system 29, the back pressure increased from 15 to 79 bars, a displacement of the stationary phase was 24%). When equilibrium between both phases was reached and no more stationary phase was pumped out, the back-pressure stayed constant at 77 bars for both systems. Under these conditions, the injection loop was filled with 14 mL of sample (corresponding to 2.8 g of hemp extract) and loaded into the rotor. Automatic fraction collection started 5 min (10 min in the case of solvent system 29) after sample injection and lasted 80 min. The fractions were collected at a regular interval of 30 s; in total, 160 fractions (25 mL) were collected. The eluent from the outlet of the system was continuously monitored by the DAD detector, and chromatograms were recorded at 220, 254, and 365 nm. Finally, the extrusion phase was initiated using an upper phase solvent as the mobile phase (600 rpm, 50 mL/min, 20 min). This was done to ensure that any residual extract was recovered from the machine.

### Purity evaluation of FCPC fractions by U-HPLC–DAD

FCPC fraction purity evaluations were performed using a U-HPLC UltiMate 3000 (Thermo Scientific, USA) equipped with a diode array detector (DAD). Analysis of all fractions was performed on a UPLC BEH C18 column (150 × 2.1 mm; 1.7 µm) (Waters) at 0.5 mL/min, 45 °C, 220 nm. The composition of mobile phases were as follows: (A) 20 mM ammonium formate in water, pH 3.2, (B) 0.15% formic acid (*v/v*) in acetonitrile. The total run time of the method was 16 min, and the injection volume was 3 µL. The gradient started at 60% of mobile phase B (0.5 mL/min) with a steep linear change to 65% of B within the first 2 min, followed by a gradual change to 70% of B (in 6 min), another gradual change to 80% of B (in 2 min), and final gradual change to 95% of B (in 3 min). At the end, the flow rate was increased to 0.6 mL/min for 1 min, and the column was then reconditioned to the initial conditions for 2 min.

The crude purity of compounds was calculated as the percentage of the peak area of the target peak as a proportion of the total integrated area throughout the chromatogram.

### U-HPLC-HRMS/MS analysis of hemp extract and its fractions

We used U-HPLC-HRMS/MS not only for the determination of phytocannabinoids in both phases by the determination of partition coefficients, but also for analysis of individual FCPC fractions. An aliquot of 100 μL was taken from every second fraction, transferred to a vial and diluted with 900 μL of ethanol prior to further analysis.

Our earlier published ISO 17025 accredited UHPLC-HRMS/MS employing internal standards Δ^9^-THC-D3, Δ^9^-THCA-D3, CBD-D3, and CBDA-D3 for quantification [24] is briefly described in the paragraph below.

For chromatographic separation, an Acquity UPLC BEH C18 reverse phase analytical column (100 × 2.1 mm; 1,7 µm, Waters) was used. Mobile phases were as follows: (A) 95:5 water–methanol (*v/v*) and (B) 65:30:5 isopropanol–methanol–water (*v/v/v*), both A and B with 5 mM ammonium formate and 0.1% formic acid (*v/v*). The total run time of the method was 19 min, and the injection volume was 3 µL. The gradient started at 5% of mobile phase B (0.3 mL/min) with a steep linear change to 60% of B in the first minute, followed by a gradual change to 70% of B (in 10 min) and another steep change to 100% B (in 0.5 min) simultaneously with a flow rate increase to 0.4 mL/min. The column was then washed with 100% B for 5 min and reconditioned to initial conditions for 2.5 min.

A high-resolution mass spectrometric detector was operated in full-scan MS acquisition mode followed by parallel reaction monitoring (PRM). Positive/negative electrospray ionization (ESI ±) parameters were as follows: auxiliary gas temperature 300 °C; sheath/aux gas (N_2_) flow 45/10 arb. u.; spray voltage: 3.5 kV; S-lens RF level 55. For the full-scan MS acquisition mode, the detection conditions were resolution 70,000 full width at half maximum (FWHM); scan range 200–1000 m*/z*; automatic gain control (AGC) target 2e5; and maximum inject time (maxIT) 50 ms. The conditions for PRM were as follows: (i) resolution 17,500 FWHM, (ii) scan range 50 –*m/z* of fragmented analyte (+ 10 m/z), (iii) AGC target 2e5, (iv) maxIT 50 ms, (v) isolation window width 1 m*/z*, and (vi) normalized collision energy (NCE) 28, 35, and 42%. The exact masses (mass window 5 ppm) of the target analytes and their fragment ions are summarized in Table 4. For data processing, we used Xcalibur 4.0 SW (Thermo Scientific, USA).

**Table 4:** Overview of the target analytes and exact masses (m/z) of their precursor and fragment ions used for the HRMS/MS target analysis of phytocannabinoids

No	Abbreviation*	Molecular formula	RT (min)	Exact masses (*m/z*) of ions**			
				[M + H] +	[M-H]-	Fragment 1	Fragment 2
1	CBD	C_21_H_30_O_2_	4.84	**315.2319**	313.2173	193.1223	259.1693
2	CBDA	C_22_H_30_O_4_	4.59	359.2217	**357.2071**	339.1966	245.1547
3	∆^8^-THC	C_21_H_30_O_2_	7.50	**315.2319**	313.2173	259.1693	241.1216
4	∆^9^-THC	C_21_H_30_O_2_	6.97	**315.2319**	313.2173	193.1223	259.1693
5	∆^9^-THCA-A	C_22_H_30_O_4_	9.80	359.2217	**357.2071**	313.2173	245.1547
6	CBG	C_21_H_32_O_2_	4.65	**317.2475**	215.2330	193.1223	137.1325
7	CBGA	C_22_H_32_O_4_	4.87	361.2373	**359.2228**	341.2122	315.2330
8	CBL	C_21_H_30_O_2_	7.77	**315.2319**	313.2173	235.1687	193.1220
9	CBLA	C_22_H_30_O_4_	11.23	359.2217	**357.2071**	339.1972	313.2177
10	CBDV	C_19_H_26_O_2_	3.49	**287.2006**	285.1860	231.1374	165.0907
11	CBDVA	C_20_H_26_O_4_	3.48	331.1904	**329.1758**	311.1657	217.1236
12	THCV	C_19_H_26_O_2_	4.54	**287.2006**	285.1860	231.1374	165.0907
13	THCVA	C_20_H_26_O_4_	6.23	331.1904	**329.1758**	283.1704	217.1234
14	CBN	C_21_H_26_O_2_	6.08	**311.2006**	309.1860	293.1885	241.1216
15	CBNA	C_22_H_26_O_4_	8.35	355.1904	**353.1758**	309.1864	279.1362
16	CBC	C_21_H_30_O_2_	8.95	**315.2319**	313.2173	259.1693	233.1531
17	CBCA	C_22_H_30_O_4_	10.74	359.2217	**357.2071**	339.1970	313.2176

*Analytes are listed in Table 1. **Exact masses of the most abundant precursor ions are highlighted in bold

## Results and discussion

As described in the Introduction, the occurrence of even traces of Δ^9^-THC/ Δ^9^-THCA-A in products prepared from insufficiently purified natural CBD may pose a risk to consumers, since ARfD, 1 μg/kg, estimated by EFSA [7], is low and can easily be exceeded. In the following paragraphs, we describe an FCPC strategy aimed at developing a rapid and effective, single-step procedure, thus ensuring safe products that do not violate legislation related to psychotropic substances. We used hemp extracts rich in CBDA/CBD, as characterized in Table 1.

### FCPC separation

The crucial step in the development of the method for a successful fractionation by FCPC is the selection of a suitable two-phase solvent system. The optimization strategy was based on the consideration of important criteria that govern the fractionation process, the partition coefficient (K_D_) of the compound of interest being one of them. Ideally, the K_D_ should be in the range 0.5 to 3 [19]. If the K_D_ value is too small (< 0.5), the analyte will not be retained in the system and will be eluted too early with poor resolution, while a higher K_D_ value (> 3) would result in broad peaks and a prolonged elution time. Another important criterion that had to be considered was the value of the separation factor α. As long as its value is 1.5 or higher, the adjacent chromatographic zones should be completely separated [25].

As any information on the FCPC fractionation of complex hemp extracts applicable for CBD isolation was available in the scientific literature, the partition coefficients K_D_ and the separation factors α were calculated for 38 different solvent systems (Table S1 and Table 3). The choice of solvent system composition was based on principles described in previous studies focused on counter-current chromatography employing biphasic solvent systems for natural products separation [19, 22, 26–29]. So-called ARIZONA solvent systems, which were mainly considered in planning of our experiments, consists of different ratios of n-heptane/ethyl acetate/methanol/water [30]. In our study, the composition of some of our solvent systems was modified by replacing n-heptane by n-pentane or n-hexane and methanol by ethanol (see Table 2).

**Fig. 1:**
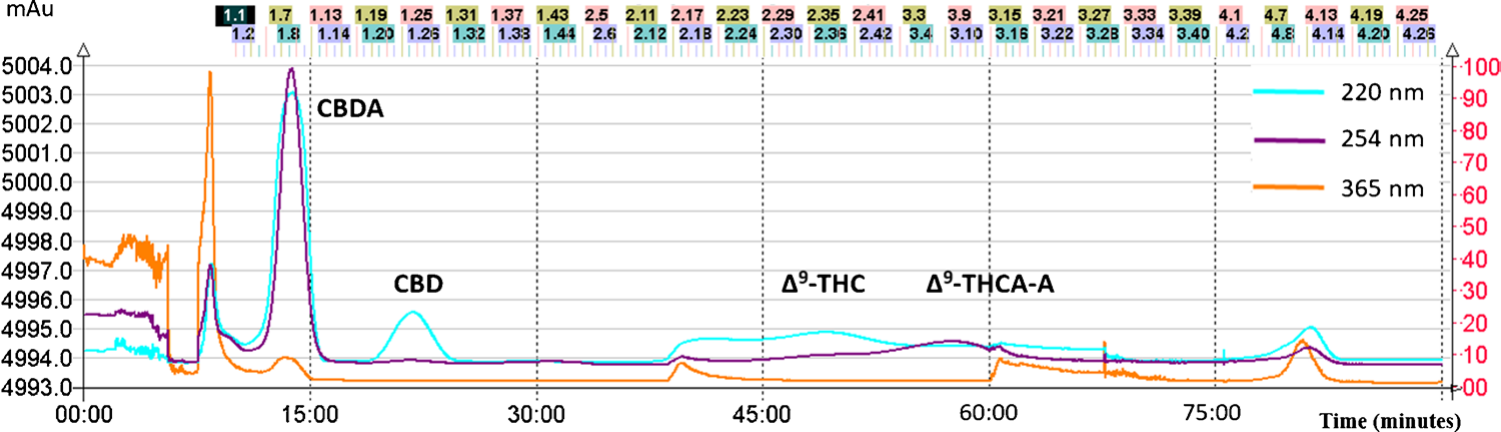
DAD chromatogram recorded at 220, 254, and 365 nm of the continuously monitored eluent from the FCPC system output for solvent system 7 consisting of n-heptane/ethyl acetate/ethanol/water (1.5:0.5:1.5:0.5, *v/v/v/v*). The fractions were collected from 5 to 85 min at regular intervals of 30 s (the identification number of each fraction is indicated at the top of the figure)

**Fig. 2:**
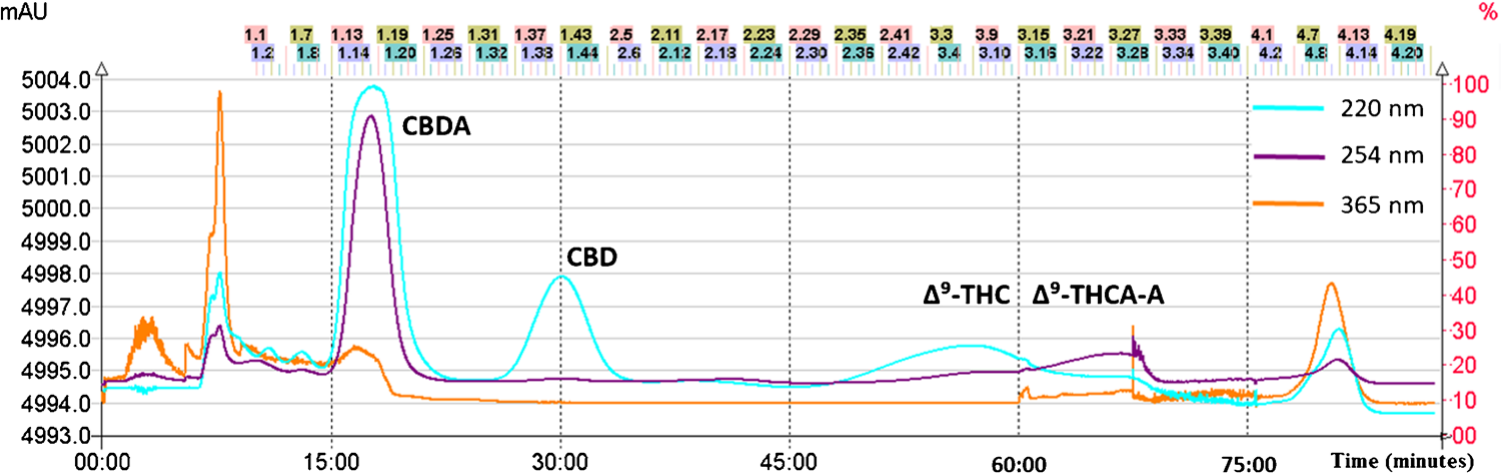
DAD chromatogram recorded at 220, 254, and 365 nm of the continuously monitored eluent from the FCPC system output for solvent system 29 consisting of n-hexane/ethyl acetate/ethanol/water (1.5:0.5:1.5:0.5, v/v/v/v) + 0.1% formic acid. The fractions were collected from 10 to 90 min at regular intervals of 30 s (the identification number of each fraction is indicated at the top of the figure)

Based on the separation criteria above, three potential biphasic solvent systems, 7, 26, and 29, were identified as promising for follow-up experiments. As the calculated values of K_D_ and α for the major analytes, CBD/CBDA and Δ^9^-THC/Δ^9^-THCA-A were almost identical for systems 7 and 26; only the first of them (without formic acid addition) was selected. Under these conditions, systems 7 and 29 were further considered as the best candidates for the separation process. As shown in Figs. 1 and 2, both systems enabled effective CBD and CBDA separation from other major phytocannabinoids present in samples; nevertheless, when using system 7 containing n-heptane instead of n-hexane, a fairly faster separation process was achieved. Moreover, n-heptane is considered to be less toxic than n-hexane [31, 32]. Based on experimental data for solvent system 7 mentioned in paragraph “FCPC fractionation procedure” and calculated K_D_ for CBD and CBDA (Table S1), the solute retention volume (V_r_) and thus elution times of analytes can be theoretically calculated using following equations [26]:

$${V}_{s}={V}_{c}-{V}_{m}$$

$${V}_{r}={V}_{m}+{K}_{D}*{V}_{s}$$

in which *V*_m_ is the mobile phase volume inside CPC column (240 mL), *V*_c_ is the maximum column volume (1000 mL), and *V*_s_ (760 mL) is the stationary phase volume. Theoretical calculated V_r_ values are 924 mL for CBDA (K_D,CBDA_ = 0.90) and 1342 mL for CBD (K_D,CBD_ = 1.45), corresponding to the theoretical elution times (with constant flow rate 50 mL/min) 18.5 and 26.8, respectively. Based on experimental data (Fig. 1), the elution times of CBDA and CBD were slightly lower, 14.5 and 22.5. This small discrepancy between theoretical value and experimental data can be explained by a specific feature of the instrument (e.g., its internal volume plus connecting tubing).

**Fig. 3:**
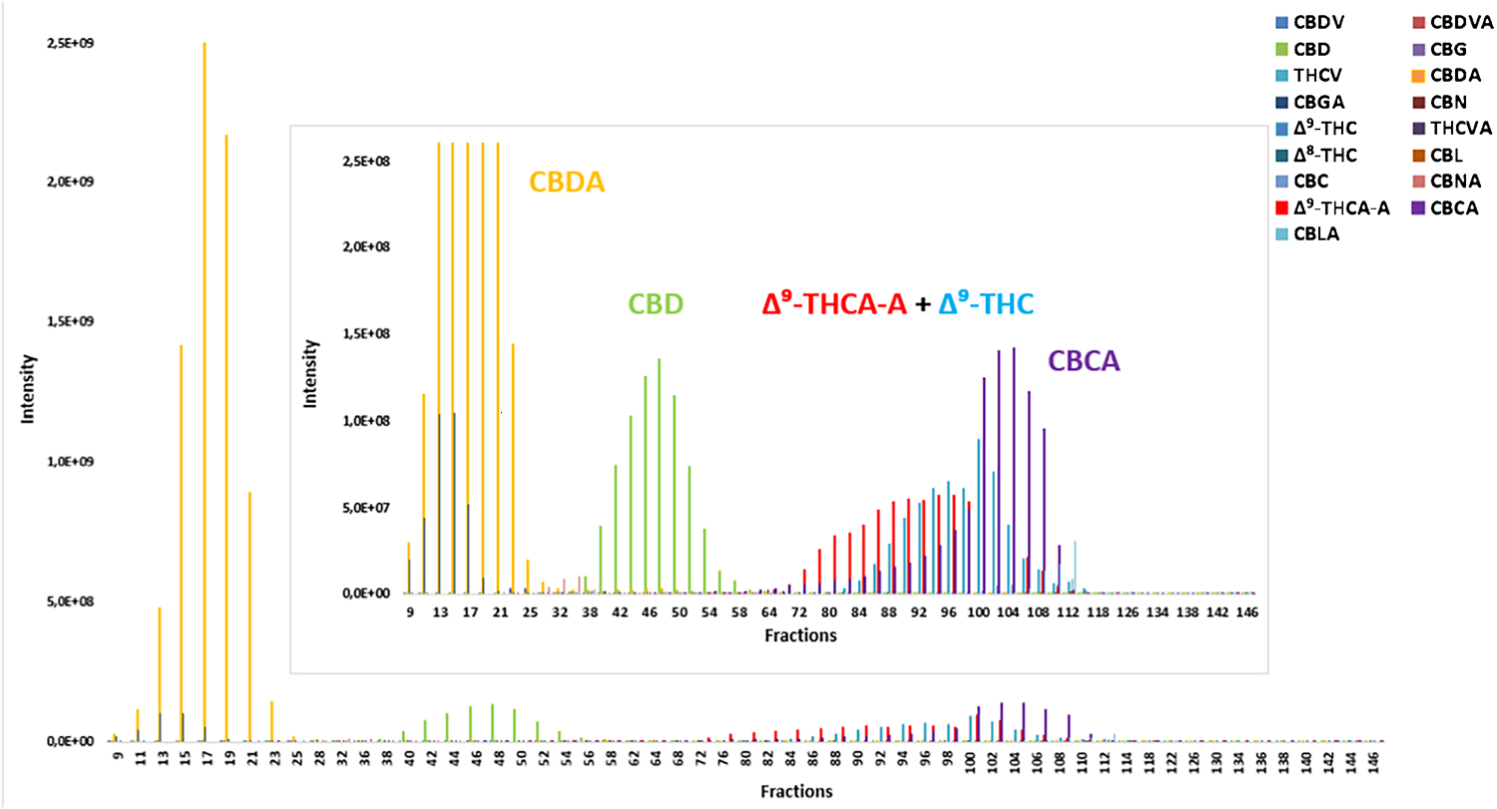
FCPC elution profiles (peak areas) of major phytocannabinoids contained in hemp extract (17 target analytes determined in each fraction using U-HPLC-HRMS/MS); solvent system 7 consisting of n-heptane/ethyl acetate/ethanol/water (1.5:0.5:1.5:0.5, v/v/v/v), 2.8 g equivalent of original extract loaded into FCPC system

Considering a limited potential of UV detection to enable confirmation/identification of compounds present in individual collected fractions (Fig. 1); therefore, U-HPLC-HRMS/MS technique was used for their analysis. Figure 3 illustrates not only elution profile of the major phytocannabinoids, the separation of which was targeted, but also the presence of other co-eluting phytocannabinoids is documented here. As shown, CBDA was eluted in fractions 10 to 24, CBD then in fractions 38 to 52. The other phytocannabinoids (with the exception of co-eluting CBGA with CBDA) and, importantly, including psychotropic compounds from the tetrahydrocannabinol group (Δ^9^-THC, Δ^9^-THCA-A), were reliably separated from CBDA and CBD and eluted in fractions from 72 to 108.

**Fig. 4:**
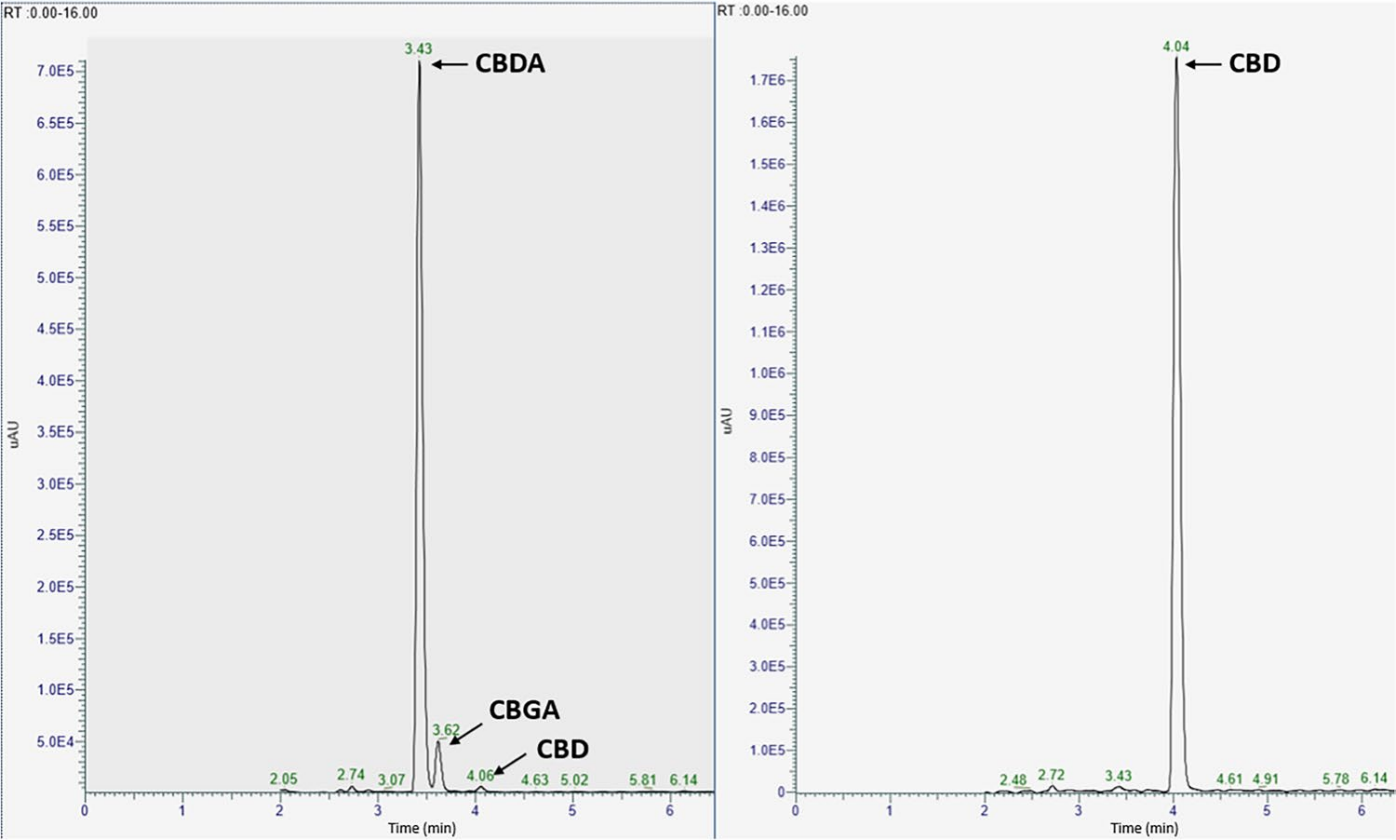
UHPLC-DAD chromatograms of the CBDA pool (fractions 10–24) and the CBD pool (fractions 38–52) recorded at 220 nm

In the first phase, to assess the crude purity of isolated products, the CBDA and CBD content was estimated in the collected fractions — pools (CBDA and CBD pools consisted of fractions 10–24 and 38–52, respectively) using U-HPLC-DAD (see Fig. 4). However, this purity evaluation should be regarded as approximate due to limitations of DAD detection, which offers lower selectivity compared to MS. It is worth noting that UV detection is commonly employed under routine conditions and is also presented in certificates provided by producers of phytocannabinoid analytical standards. The purity of CBDA and CBD pools determined in this way was 92.5% and 98.0%, respectively, and was calculated as the proportion of a particular peak area relative to the total integrated area throughout the chromatogram (= 100%).

To obtain more detailed information on the phytocannabinoids that occur in the pools, a U-HPLC HRMS/MS target analysis involving 17 analytes was performed; the results are summarized in Table 5. It should be noted that the purity determined by this alternative analytical strategy was not identical to the U-HPLC-DAD results. Higher purities for both CBDA and CBD were obtained by U-HPLC-HRMS/MS (individual calibration standards used for quantification). Concerning the CBDA pool, its purity (calculated based on the weight of CBDA and the total weight of the CBDA fraction) was 95.1%. Among the remaining “impurities,” 4.6% were co-eluting CBGA > CBD > CBG. In addition, traces of CBDV and CBDVA were also detected. In the CBD pool, the CBD content (calculated in the same way as for CBDA) was 98.9%, with the remaining 1.1% mainly comprising CBDA accompanied by a small amount of THCV and traces of CBGA, CBNA, and CBG.

**Table 5:** The total content (in mg) of 17 phytocannabinoids in pooled CBDA and CBD fractions obtained under conditions of experiment

Phytocannabinoid	CBDA pool (fractions 11–24) mg of analyte in pool*	CBD pool (fractions 38–54) mg of analyte in pool*
CBDA	902	1.13
CBD	9.74	151
CBG	5.39	0.05
CBGA	25.9	0.07
CBDVA	1.07	< 0.01
CBDV	1.33	< 0.01
THCV	< 0.01	0.21
CBNA	< 0.01	0.06

*The content of Δ^*9*^*-THC****,**** Δ*^*8*^*-THC, Δ*^*9*^*-THCA-A, CBL, CBN, CBC, CBLA, CBCA, THCVA* < *0.01*

The FCPC recovery of CBDA and CBD in collected fractions, calculated on the basis of mass balance, was 94.8% and 89.3%, respectively. If a narrower CBDA fraction (16–24, i.e., 18–22.5 min) was collected to eliminate the co-eluting CBGA, then the recovery of this compound would be lower, around 60%. Nevertheless, the purity would increase up-to 97%. However, based on current knowledge, the co-occurrence of CBGA in the CBDA fraction should not pose a major problem as it is a precursor of CBG, which exhibits a wide range of beneficial biological activities, including anti-inflammatory, antibacterial, and antifungal activities, regulation of redox balance, and neuromodulatory effects [33]. Although CBDA has also been reported to exhibit some biological activities, including antibacterial activity and anti-nausea/emetic effects [34, 35], this compound (in most hemp extracts, the major phytocannabinoid) is usually used to obtain CBD by thermal conversion as the product of its decarboxylation [36]. In this way, the yield of desired CBD could be increased, although thermally induced conversion of CBDA is not stoichiometric and other by-products are formed [37, 38]. In other earlier studies related to the separation of phytocannabinoids by centrifugal partition chromatography (CPC), the possible phytocannabinoids co-elution/purification issues were resolved either by pre-separation of acidic and neutral cannabinoids (acidic analytes were retained from hexane extract on acid-washed sea sand filter) [14] or by pH-zone-refining based on trifluoroacetic acid as retainer of acidic phytocannabinoids in organic stationary phase, while triethylamine was used as elute in the aqueous mobile phase [15]. Sophisticated although rather demanding to develop trapping multiple dual mode (MDM) approach in a flow-reversal liquid-liquid chromatography (LLC), operating mode was shown to be effective for the isolation of some minor phytocannabinoids. Nevertheless, the decarboxylated “green” extract was used, and, therefore, separation of acidic forms from neutral ones was not necessary to resolve [16]. The purpose of our study was rather different, and the key objective was to implement a simple, “dilute and shoot” procedure for the fast isolation of CBD/CBDS free of psychotropic compounds from the tetrahydrocannabinol group.

As the purity of CBD/CBDA fractions was  < 100%, their further UHPLC-HRMS investigation was performed. Besides the 17 major phytocannabinoids, for which analytical standards were available, thus could be quantified, minor phytocannabinoids and other bioactive compounds such as terpenoids, flavonoids, stilbenoids, alkaloids, and phenolic amides possibly present in *Cannabis sativa *L. extract and co-eluting in CBD/CBDA fractions were searched [39, 40]. In-house created spectral library involving altogether 754 secondary metabolites that have been identified in *Cannabis sativa* L. plants and reported in the scientific literature [39, 41–44] was used for target screening of respective accurate *m/z* values, both in positive (protonated molecules) and negative ionization mode (deprotonated molecules). When considering signals with areas  > 10e^5^, i.e., 2–3 orders of magnitude lower (Fig. 3) compared to those of CBDA and CBD, then 56 and 25 compounds were detected, respectively. An example of relative signal areas in pooled CBDA and CBD fractions are shown in Figure S1 and Figure S2. Relative concentrations of these compounds can be roughly estimated as no significant differences exist within the neutral and/or acidic phytocannabinoids for which standards are available. However, neither investigation of matrix effects nor identification of detected compounds could be performed. In the latter case, this was due to the poor quality of the fragmentation spectra and the unavailability of the libraries.

In spite of the low concentration of co-eluted compounds, their impact on the biological activity of the isolated fraction still cannot be excluded. To obtain 100% pure products, additional fractionation would be necessary using another FCPC solvent system selected based on K_D_ and α values summarized in Table S1. In this context, discussions on the biological activities of “full-spectrum” *Cannabis* extracts are ongoing, specifically where preparations are intended for the treatment of various diseases [45]. Several authors mention “entourage effects” [46]; however, various contradictory opinions have been published [47, 48]. In any case, the biological effects of pure phytocannabinoids such as CBD are better understood and more predictable; therefore, minimization of co-eluted metabolites is always desirable. Needed to mention that some phytocannabinoid producers declare the superiority of molecular distillation technique in terms of achievable high purity (as high as 99% and even more) of isolated products. Unfortunately, no scientific studies focused on parameters of this process have been published, yet. Nevertheless, some producers of distillation equipment emphasize the importance of proper conditions control (vacuum, temperature); otherwise, phytocannabinoids thermal degradation (mainly decarboxylation and/or oxidation) during the fractionation process might take place.

## Conclusions

In this study, a simple “dilute-and-shoot” FCPC procedure has been developed for the isolation of CBDA and CBD; the key outcomes are summarized in the following points:

- The selection of a solvent system that allowed an effective separation of target compounds, CBDA and CBD, from Δ^9^-THC, Δ^9^-THCA-A, and other psychotropic phytocannabinoids, was possible based on partition coefficients (K_D_) and separation factors (α). Data for their calculation were obtained by testing the partition of 17 phytocannabinoids contained in hemp extract in 38 solvent systems.
- In line with the prediction that took into account experimental K_D_ and α values, n-heptane/ethyl acetate/ethanol/water (1.5:0.5:1.5:0.5, *v/v/v/v*) biphasic solvent was shown to be suitable for the isolation of CBDA and CBD; respective fractions had 95.4 and 99.0% (w/w), respectively. The presence of Δ^9^-THC, Δ^9^-THCA-A in these fractions was not detected.
- Traces of secondary metabolites of hemp with phytocannabinoid-like structures and other biologically active compounds were detected, in both the CBDA and CBD fractions, using targeted UHPLC-HRMS screening against an in house-created spectral library.

In conclusion, the main advantage of CPC is its generally easy scaling-up to an industrial level, once the method has been developed. The large-scale separation significantly reduces the costs, mainly because of lower solvent consumption per 1 g of pure compound and the absence of expensive solid stationary phase. As regards phytocannabinoids isolation, developed CPC method is a great alternative to other purification techniques used in industry, such as molecular distillation.

## Supplementary Information

Below is the link to the electronic supplementary material.

Supplementary file1 (DOCX 229 KB)
