# Supplementary material for: Effective isolation of cannabidiol and cannabidiolic acid free of psychotropic phytocannabinoids from hemp extract by fast centrifugal partition chromatography
Source: Anal Bioanal Chem. 2023 Jun 29;415(19):4827–37. doi: 10.1007/s00216-023-04782-9 (PMC10352166; doi:10.1007/s00216-023-04782-9)
Supplement: Supplementary file 1 — Supplementary file1 (DOCX 229 KB) [file 216_2023_4782_MOESM1_ESM.docx]

**Supplementary material** (Figures S1 and S2 should be printed in colour):

**Table S1.** Overview of the calculated partition coefficients (KD) for 38 solvent systems. *

| **Solvent system** | **CBDA** | **CBD** | **Δ^9^-THCA-A** | **Δ^9^-THC** | **CBDVA** | **CBDV** | **CBG** | **CBGA** | **CBN** | **THCVA** | **CBNA** | **CBC** | **CBLA** | **CBCA** | **CBL** | **THCV** |
| --- | --- | --- | --- | --- | --- | --- | --- | --- | --- | --- | --- | --- | --- | --- | --- | --- |
| **1** | 10.6 | 63.9 | 71.2 | 92.7 | 89.1 | 122.3 | 95.6 | 26.3 | 94.1 | 999 | 11.6 | 75.8 | 84.3 | 117.1 | 999 | 0.00 |
| **2** | 3.31 | 16.1 | 13.2 | 29.3 | 9.13 | 16.5 | 27.3 | 6.31 | 26.9 | 999 | 2.62 | 26.6 | 17.1 | 31.6 | 0.00 | 0.00 |
| **3** | 1.11 | 2.64 | 4.17 | 8.22 | 0.61 | 1.84 | 2.10 | 1.10 | 5.11 | 999 | 3.12 | 10.6 | 4.69 | 17.3 | 0.00 | 0.00 |
| **4** | 0.30 | 0.18 | 0.41 | 0.34 | 0.01 | 0.12 | 999 | 0.12 | 0.22 | 0.44 | 0.86 | 0.34 | 0.41 | 0.42 | 0.22 | 0.00 |
| **5** | 6.34 | 31.9 | 39.9 | 51.8 | 47.2 | 38.9 | 53.5 | 17.4 | 57.6 | 0.00 | 7.40 | 51.3 | 49.9 | 128.8 | 0.11 | 1.82 |
| **6** | 2.74 | 9.81 | 10.8 | 18.3 | 6.54 | 10.0 | 17.6 | 4.60 | 18.4 | 0.00 | 4.80 | 20.3 | 15.7 | 44.1 | 0.00 | 999 |
| **7** | 0.90 | 1.45 | 2.14 | 3.11 | 0.52 | 1.11 | 1.04 | 0.73 | 2.41 | 999 | 4.00 | 3.71 | 2.88 | 3.19 | 0.00 | 999 |
| **8** | 5.89 | 29.8 | 31.1 | 45.4 | 46.4 | 45.1 | 52.5 | 16.5 | 43.7 | 999 | 4.30 | 48.2 | 39.4 | 32.8 | 999 | 999 |
| **9** | 3.71 | 17.9 | 23.4 | 38.1 | 12.3 | 13.9 | 36.7 | 8.43 | 30.7 | 0.00 | 5.31 | 41.2 | 31.3 | 76.8 | 0.00 | 0.00 |
| **10** | 1.29 | 2.14 | 3.06 | 4.11 | 0.74 | 1.54 | 1.34 | 0.94 | 2.81 | 999 | 3.02 | 4.40 | 3.55 | 8.47 | 999 | 999 |
| **11** | 0.50 | 0.51 | 1.44 | 1.12 | 0.11 | 0.23 | 0.24 | 0.33 | 0.72 | 999 | 4.34 | 1.40 | 1.39 | 2.54 | 0.00 | 999 |
| **12** | 2.71 | 11.4 | 17.2 | 30.1 | 5.04 | 8.61 | 18.8 | 4.80 | 23.6 | 999 | 7.03 | 32.2 | 24.1 | 62.5 | 999 | 999 |
| **13** | 4.20 | 17.7 | 20.3 | 32.1 | 16.2 | 18.8 | 38.2 | 9.71 | 35.2 | 12.4 | 4.94 | 31.6 | 25.2 | 43.1 | 15.8 | 0.00 |
| **14** | 5.91 | 33.4 | 38.2 | 56.1 | 45.2 | 53.9 | 61.3 | 12.8 | 62.5 | 17.8 | 7.90 | 50.2 | 41.6 | 154.8 | 0.00 | 0.00 |
| **15** | 2.59 | 9.01 | 10.3 | 16.8 | 5.34 | 8.74 | 14.9 | 4.32 | 16.6 | 999 | 3.81 | 18.6 | 13.2 | 19.7 | 999 | 0.00 |
| **16** | 7.71 | 40.6 | 43.3 | 62.2 | 56.0 | 36.9 | 57.8 | 15.6 | 73.8 | 0.0 | 9.36 | 64.4 | 50.8 | 119.7 | 0.00 | 999 |
| **17** | 6.11 | 38.0 | 48.0 | 61.1 | 26.8 | 36.9 | 65.4 | 24.1 | 60.6 | 0.0 | 999 | 63.7 | 54.6 | 82.6 | 0.00 | 1.12 |
| **18** | 1.33 | 2.70 | 5.44 | 7.52 | 0.88 | 1.81 | 2.22 | 1.30 | 5.44 | 999 | 5.35 | 9.81 | 7.60 | 20.3 | 999 | 999 |
| **19** | 8.12 | 47.1 | 54.6 | 86.0 | 72.0 | 57.5 | 84.0 | 22.0 | 81.4 | 0.0 | 14.5 | 61.7 | 65.7 | 182.0 | 0.0 | 999 |
| **20** | 6.10 | 32.2 | 33.9 | 51.0 | 45.0 | 47.3 | 57.7 | 15.7 | 48.3 | 999 | 7.77 | 50.4 | 43.2 | 62.4 | 5.90 | 0.00 |
| **21** | 3.21 | 12.4 | 13.0 | 20.2 | 12.1 | 13.9 | 22.8 | 6.12 | 22.9 | 7.45 | 7.50 | 23.3 | 16.9 | 32.0 | 0.00 | 0.00 |
| **22** | 1.31 | 2.74 | 5.22 | 8.30 | 0.77 | 1.79 | 2.20 | 1.22 | 5.44 | 999 | 8.41 | 11.0 | 9.64 | 21.1 | 999 | 0.00 |
| **23** | 0.41 | 0.19 | 0.61 | 0.42 | 0.11 | 0.08 | 0.09 | 0.21 | 0.34 | 1.23 | 1.62 | 0.44 | 0.49 | 0.59 | 0.00 | 999 |
| **24** | 3.94 | 15.3 | 16.3 | 25.7 | 22.5 | 18.2 | 27.7 | 9.00 | 27.2 | 13.4 | 5.74 | 23.5 | 19.9 | 24.3 | 21.2 | 999 |
| **25** | 2.51 | 7.44 | 8.20 | 13.4 | 5.85 | 8.80 | 13.1 | 4.14 | 14.1 | 15.5 | 3.23 | 14.1 | 10.2 | 14.1 | 28.1 | 0.00 |
| **26** | 0.90 | 1.47 | 2.25 | 3.05 | 0.51 | 1.02 | 1.11 | 0.80 | 2.42 | 3.41 | 5.12 | 4.13 | 3.09 | 4.21 | 0.00 | 0.00 |
| **27** | 7.20 | 40.9 | 48.0 | 62.8 | 62.9 | 52.7 | 80.6 | 17.7 | 78.5 | 29.4 | 7.21 | 66.2 | 52.6 | 88.8 | 999 | 0.00 |
| **28** | 3.31 | 16.8 | 21.4 | 31.5 | 12.1 | 15.5 | 28.4 | 7.10 | 30.0 | 9.94 | 999.0 | 37.0 | 28.2 | 82.1 | 0.00 | 999 |
| **29** | 1.04 | 1.76 | 2.74 | 3.74 | 0.71 | 1.40 | 1.31 | 0.85 | 3.04 | 1.92 | 3.59 | 4.54 | 3.60 | 9.28 | 2.91 | 999 |
| **30** | 0.61 | 0.51 | 1.44 | 1.12 | 0.07 | 0.30 | 0.19 | 0.32 | 0.74 | 999 | 2.84 | 1.40 | 1.54 | 2.72 | 999 | 999 |
| **31** | 2.81 | 13.0 | 8.03 | 37.9 | 5.04 | 10.3 | 20.9 | 4. 86 | 30.9 | 7.14 | 4.33 | 42.8 | 38.6 | 999 | 0.00 | 0.00 |
| **32** | 4.54 | 23.6 | 27.7 | 44.1 | 18.9 | 21.3 | 40.4 | 9.29 | 44.3 | 999 | 8.54 | 46.0 | 35.1 | 104.9 | 999 | 0.00 |
| **33** | 12.6 | 100.6 | 50.0 | 205.1 | 90.3 | 276.6 | 158.1 | 31.8 | 174.6 | 66.0 | 10.9 | 186.9 | 119.7 | 999 | 0.00 | 999 |
| **34** | 2.74 | 11.4 | 14.9 | 23.2 | 5.51 | 9.92 | 15.2 | 4.60 | 20.9 | 999 | 5.34 | 25.2 | 19.5 | 36.7 | 999 | 999 |
| **35** | 6.18 | 35.2 | 39.5 | 51.9 | 50.5 | 35.5 | 60.0 | 18.3 | 64.9 | 999 | 10.6 | 65.8 | 42.9 | 88.2 | 999 | 0.00 |
| **36** | 5.21 | 30.9 | 38.6 | 53.9 | 22.4 | 23.8 | 46.6 | 10.8 | 49.2 | 999 | 9.51 | 48.0 | 49.0 | 999 | 0.00 | 0.00 |
| **37** | 1.40 | 3.21 | 6.44 | 7.60 | 1.07 | 2.16 | 2.80 | 1.54 | 6.51 | 999 | 6.40 | 12.8 | 9.74 | 21.9 | 0.00 | 999 |
| **38** | 9.11 | 56.6 | 59.3 | 81.4 | 81.8 | 192.4 | 111.5 | 32.5 | 84.2 | 999 | 7.22 | 75.7 | 80.6 | 125.3 | 0.00 | 0.00 |

* *The concentration of analyte ∆^8^-THC was below LOQ, therefore is not included in the table.*


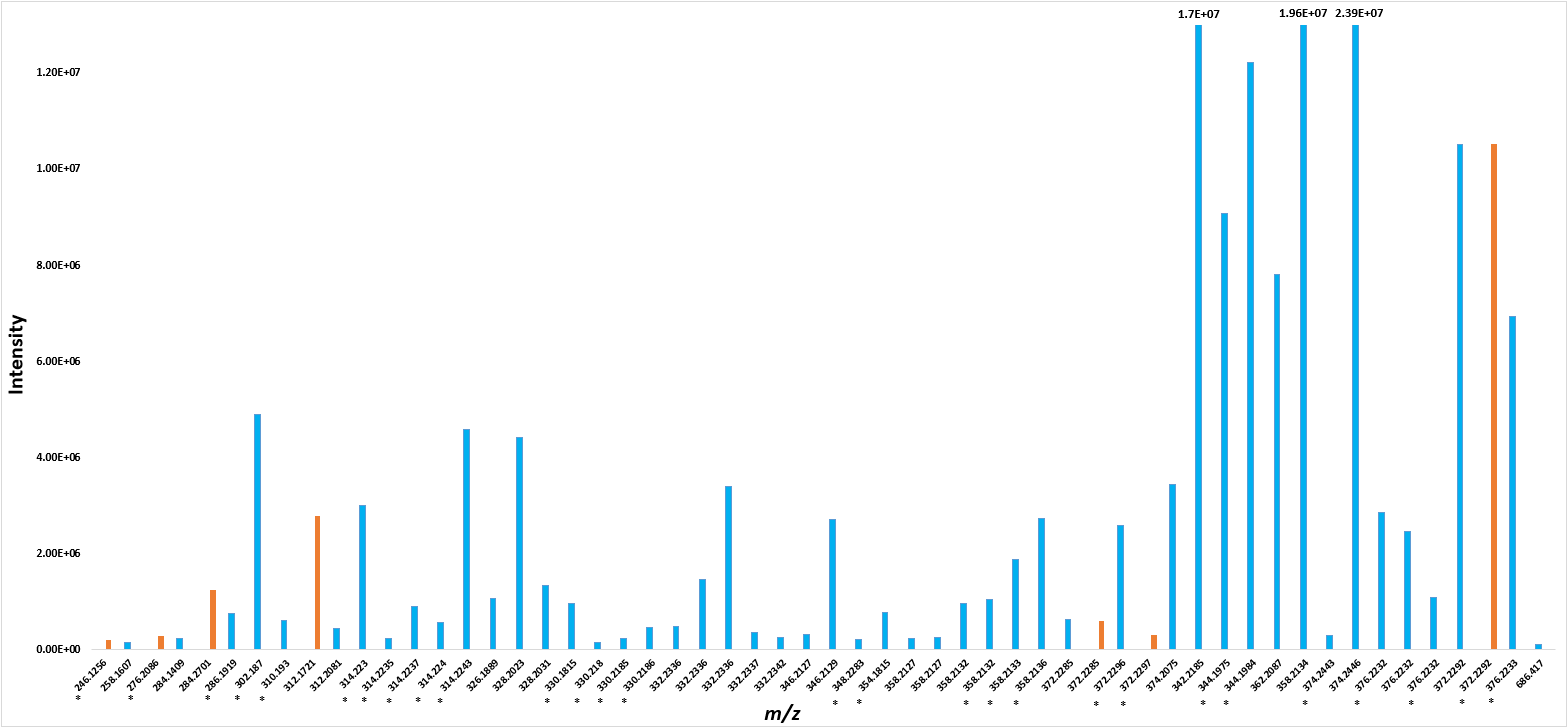


**Fig. S1:** Areas of 56 compounds detected in pooled CBDA (fractions 38-52 in **Figure 3**) target by UHPLC-HRMS/MS screening against exact masses contained in in-house spectral library (mass accuracy 5ppm); compounds with phytocannabinoids-like structure in blue, other biologically active compounds in orange, * compounds detected in ESI-, unlabelled compounds detected in ESI+.


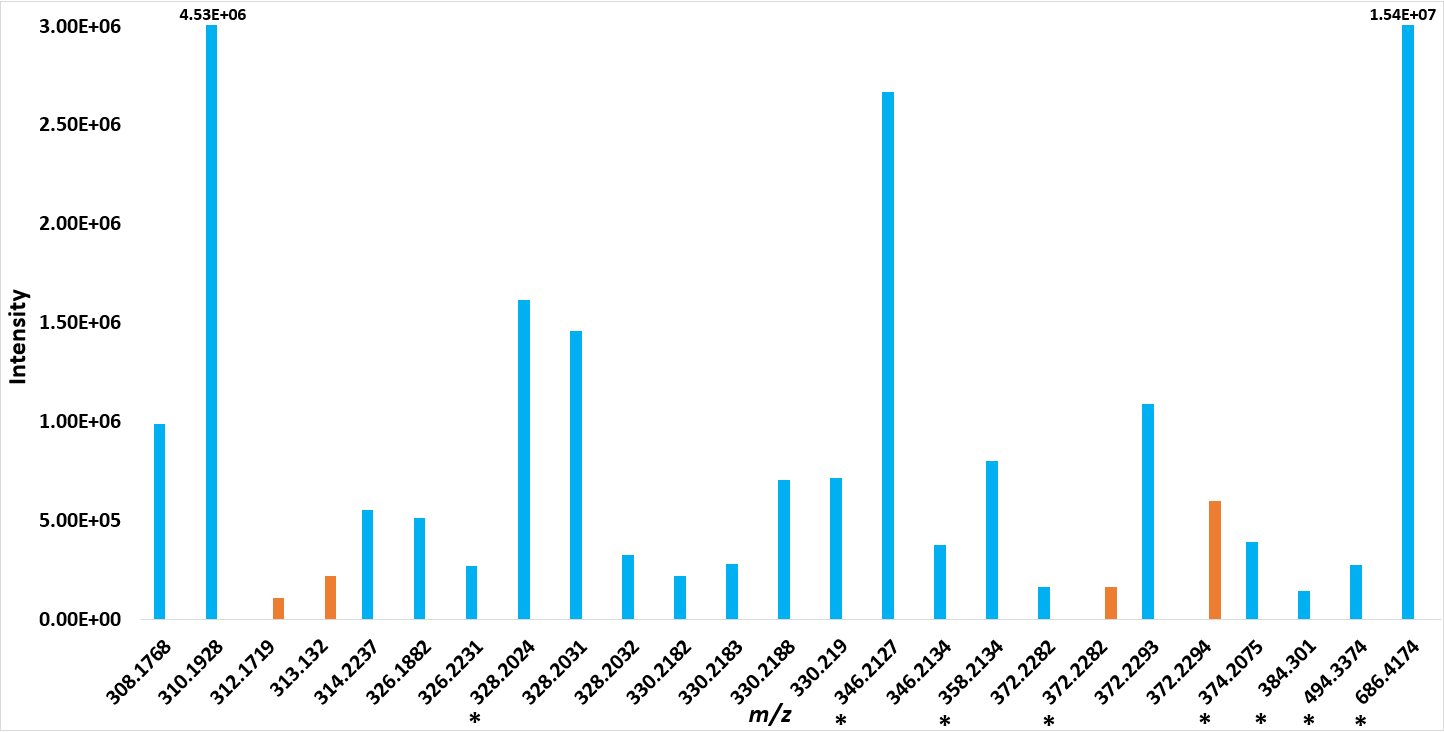


**Fig. S2:** Areas of 25 compounds detected in pooled CBD (fractions 38-52 in **Figure 3**) target by UHPLC-HRMS/MS screening against exact masses contained in in-house spectral library (mass accuracy 5ppm); compounds with phytocannabinoids-like structure in blue, other biologically active compounds in orange, * compounds detected in ESI-, unlabelled compounds detected in ESI+.


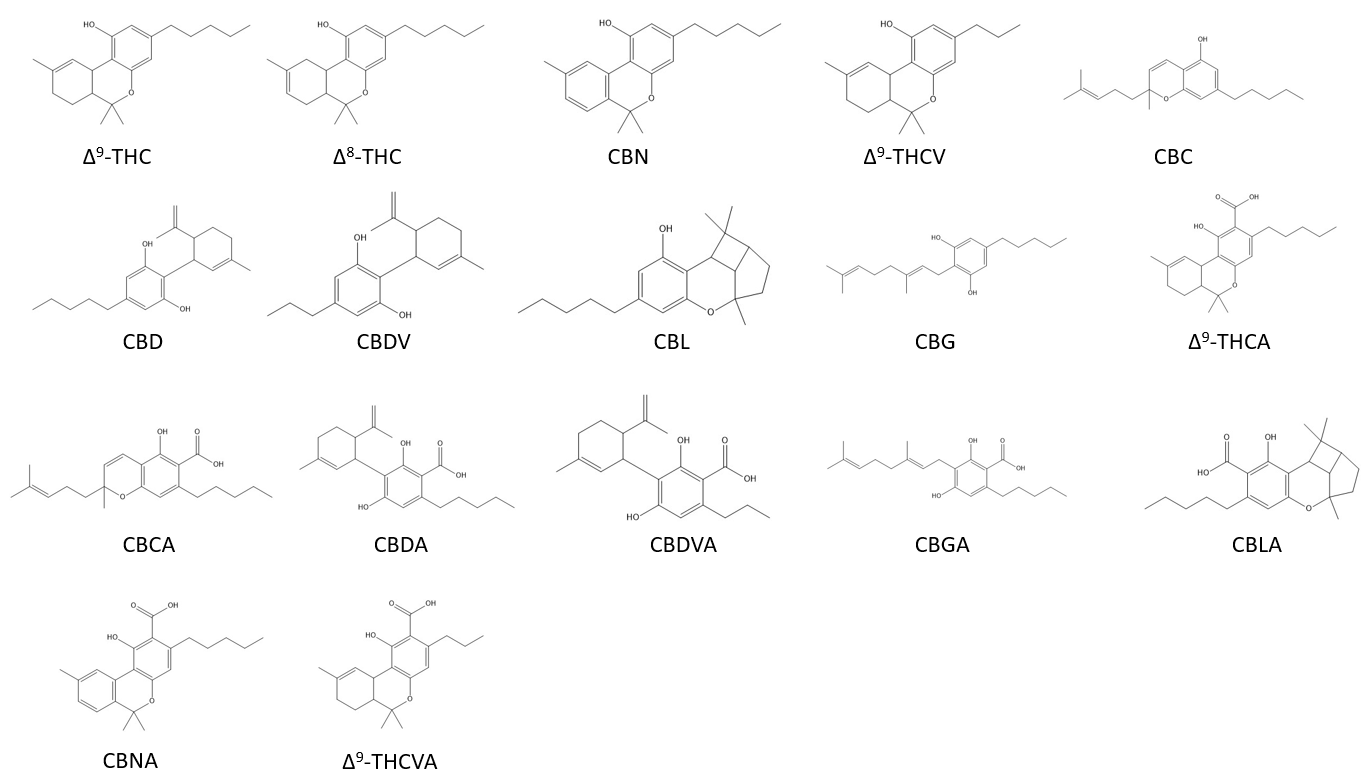


**Fig. S3:** Molecular structures of 17 phytocannabinoids used for quantitative analysis by UHPLC-HRMS/MS.
